# Prodromal stage in Lewy body dementia in a population of Argentina

**DOI:** 10.1002/alz.086436

**Published:** 2025-01-03

**Authors:** Maria Cecilia Fernandez, Waleska Berrios, Nuria Campora, Angel Golimstok

**Affiliations:** ^1^ Hospital Italiano de Buenos Aires, Buenos aires Argentina; ^2^ Churruca Visca Hospital, Buenos Aires Argentina; ^3^ Complejo Médico hospitalario Churruca Visca, buenos aires Argentina

## Abstract

**Background:**

Prodromal stage of Lewy Body Dementia (LBD) is characterized by a set of symptoms that do not yet meet the criteria for dementia. These symptoms are caused by changes in the brain that have not yet reached the threshold for triggering the disease in its complete form.

Identifying the prodromal stage of neurodegenerative diseases is crucial for early treatment and prevention of progression to a clinical stage. This study aimed to determine the clinical profile in the prodromal stage of LBD in a population of Argentina.

**Method:**

Over six months, we recruited subjects with dementia and a control group of individuals without neurocognitive disorder (NC). All participants underwent a comprehensive medical history, neuropsychological tests, and neuroimaging.

Dementia patients were divided into two groups: Group 1 consisted of patients with probable DLB according to the consensus criteria of McKeith et al. 2017. Group 2 consisted of patients with Major Neurocognitive Disorder due to Alzheimer’s Disease (AD) according to DSM V criteria.

To detect the presence of early clinical signs, we interviewed family members of patients based on central and supportive clinical signs according to Consortium‐DLB 2017 that may have been present up to 20 years before the dementia diagnosis.

**Result:**

The study included 212 participants; 56 were classified as NC, 72 in Group 1, and 84 in Group 2. No significant differences were observed in sociodemographic factors. Notably, LBD patients exhibited a higher frequency of **REM sleep behavior disorder** (12.5%), **visual hallucinations** (9.7%), and **syncope** (9.7%) compared to the other groups, with statistical significance (p<0.01). Other variables did not show significant differences.

**Conclusion:**

In this population with LBD, **visual hallucinations, syncope, and REM sleep behavior disorder** may occur more than 20 years before the onset of dementia and constitute signs of the prodromal stage. Encouraging the search for these symptoms is essential since they are not frequently questioned in patients with mild cognitive complaints.

